# Enhanced Enzymatic Activity of Yeast-like Fungi Responsible for Onychomycosis in Renal Transplant Recipients

**Published:** 2006-02

**Authors:** Jolanta Weglowska, Adam Reich, Bronisława Walów, Jacek C. Szepietowski

**Affiliations:** 1 *Department of Dermatology, Regional Hospital, Wrocław, Poland;*; 2 *Department of Dermatology, Venereology and Allergology, University of Medicine, Wrocław, Poland*

**Keywords:** onychomycosis, renal transplantation, enzymatic activity, yeasts

## Abstract

**Background:**

Renal transplant recipients (RTR) are regarded as a group especially predisposed to onychomycosis. The exact mechanism of increased frequency of onychomycosis in RTR is however not fully understood.

**Objectives:**

This study was undertaken to evaluate activity of hydrolitic enzymes of fungi most commonly causing fungal nail infections in RTR and to compare it with enzymatic activity of the same fungi isolated from lesional nails in immunocompetent patients.

**Material and methods:**

28 strains of yeast-like fungi cultured from lesional nails in RTR and 25 strains of yeasts isolated from changed nails in immunocompetent patients were included into the study. All fungi were identified on the basis of routine mycological procedures. Activity of 19 hydrolytic enzymes was assessed by API ZYMÒ test (bioMerieux).

**Results:**

Fungi cultured from RTR showed activity of 16 out of 19 enzymes, whereas fungi isolated from immunocompetent patients only 11 out of 19 enzymes. Moreover, yeast-like fungi isolated from RTR showed higher generally higher activity of detected enzymes compared to yeast strains obtained from the lesional nails of immunocompetent patients.

**Conclusions:**

This study shows for the first time enhanced enzymatic activity of yeast-like fungi isolated from lesional nails in RTR in comparison to fungi cultured from changed nails in immunocompetent patients. It is hypothesized that this enhanced enzymatic activity may be responsible for higher incidence of onychomycosis in RTR.

## INTRODUCTION

Onychomycosis is the most frequent nail disease. It accounts for about a half of all nail abnormalities and for one third of all mycotic infection of the skin (1, 2). The frequency of onychomycosis in the highly developed countries ranges between 3% and 8% depending on the examined population (3-5). Athough onychomycosis is not a life-threatening disease it is not just a cosmetic problem as many patients with onychomycosis had significantly decreased quality of life (6-8). It is estimated that annual costs of the treatment of onychomycosis in the US healthcare system amount more than 43 million of dollars (9).

Cutaneous complications are frequently observed in renal transplant recipients (RTR). Due to immunossupressive treatment RTR are especially predisposed to different types of infections, among them fungal infections, including onychomycosis, are regarded as the most common ones (10). Recently, evaluating 216 RTR, our group has shown that 19.9% of RTR suffered from fungal nail infections (11). Yeast-like fungi appeared to be the most commonly isolated fungi from the lesional nails (47.45%), followed by molds (45.75%) and dermatophytes (only 6.8%) (11). The pathomechanism of increased frequency of onychomycosis in RTR is however not completely clear. Therefore, this study was undertaken to evaluate enzymatic activity of the most common pathogens of onychomycosis in RTR (yeast-like fungi in our studied group of RTR) and to compare it with enzymatic activity of the same fungi isolated from lesional nails in immunocompetent patients.

## MATERIAL AND METHODS

28 strains of yeast-like fungi cultured from lesional nails of 24 RTR (Candida albicans-15; Candida sp.-8 and Rhodotorula rubra-5) and 25 strains of yeasts isolated from changed nails in immunocompetent patients (Candida albicans-9; Candida sp.-7 and Rhodotorula rubra-7) were included into the study. The group of RTR consisted of 13 (54.2%) women and 11 (45.8%) men in the age between 23 and 64 years (mean 46 ± 12.1 years). Among immunocompetent subjects there were 18 (72%) females and 7 (28%) males. Their age ranged between 19 and 76 years (mean 51.2 ± 18 years). No patient demonstrated other clinical conditions, including diabetes and peripheral vascular diseases, which could predispose for the development of onychomycosis. Both immunocompromised and immunocompetent patients presented with distal and lateral subungual clinical (DLSO) type of onychomycosis. There was no significant difference in number of infected nails, extend and duration of infection between two studied groups. Identification of all fungi was based on routine mycological procedures (direct microscopy and mycological culture on different media) (12). Activity of 19 hydrolytic enzymes (alcaline phosphatase, esterase (C4), esterase lipase (C8), lipase (C14), leucine arylamidase, valine arylamidase, cystine arylamidase, trypsin, α-chymotrypsin, acid phosphatase, naphtol-AS-BI-phosphohydrolase, α-galactosidase, β-galactosidase, β-glucoronidase, α-glucosidase, β-glucosidase, n-acetyl-β-glucosamidase, α-mannosidase and α-fucosidase) was assessed by API ZYMÒ test (bioMerieux). Active strains were considered only those that demonstrated visible changes in the colour of the medium. The intensity of the colour reflected the amount of degraded substrate - the activity of enzymes was expressed in nmol of hydrolyzed substrates. Statistical analysis was performed using Statistica 6.0 for Windows Software. The data were analyzed with Mann Whitney U test or Fisher exact test were appropriate; *p* values less than 0.05 were considered significant.

## RESULTS

Yeast-like fungi isolated from the changed nails in RTR expressed activity of 16 out of 19 enzymes, whereas fungi cultured from immunocompetent patients only 11 out of 19 enzymes. The following enzymes were detected only in strains achieved from RTR: lipase (C14) (n=5), cystine arylamidase (n=19), a-chymotrypsin (n=1), a-galactosidase (n=1), a-mannosidase (n=4). None of the analyzed fungi (both in RTR and in control group) demonstrated activity of tripsin, β-glucoronidase, and α-fucosidase.

All yeast-like fungi isolated from RTR significantly more often revealed the activity of cystine arylamidase (*p*<0.0001) and β-glucosidase (*p*=0.04), and showed significantly higher enzymatic activity of esterase (C4) (*p*<0.001), esterase lipase (C8) (*p*<0.0001), leucine arylamidase (*p*=0.01), valine arylamidase (*p*<0.01), acid phosphatase (*p*<0.01), naphtol-AS-BI-phosphohydrolase (*p*<0.0001) and n-acetyl- β-glucosamidase (*p*=0.03) compared to yeast strains obtained from the lesional nails of immunocompetent patients (Table 1).

**Table 1:** Comparison of enzymatic activity between fungi isolated from renal transplant recipients and healthy controls (The significance of differences in number of active strains was assessed with Fisher exact test and the significance of differences in mean activity of enzymes with Mann-Whitney *U* test)

Enzyme		All yeast-like fungi		Candida albicans		*Candida sp*. (other than *C. albicans*)		Rhodotorula rubra	
		Number of active strains (%)	Mean activity ± SD [nmol]^a^	Number of active strains (%)	Mean activity ± SD [nmol]^a^	Number of active strains (%)	Mean activity ± SD [nmol]^a^	Number of active strains (%)	Mean activity ± SD [nmol]^a^

alcaline phosphatase	RTR	28 (100%)	11.4 ± 9.3	15 (100%)	9 ± 8.7	8 (100%)	20 ± 7.6	5 (100%)	5 ± 0
	Controls	25 (100%)	11.6 ± 12.1	8 (100%)	8.7 ± 8.8	10 (100%)	18.5 ± 15.3	7 (100%)	5 ± 0
	P	-	0.57	-	0.95	-	0.59	-	1.0
esterase (C4)	RTR	28 (100%)	32.9 ± 8.5	15 (100%)	30.7 ± 10.3	8 (100%)	32.5 ± 4.6	5 (100%)	40 ± 0
	Controls	25 (100%)	23 ± 8.4	8 (100%)	21.9 ± 10	10 (100%)	24 ± 8.4	7 (100%)	22.9 ± 7.6
	P	-	<0.001	-	<0.05	-	0.02	-	<0.01
esterase lipase (C8)	RTR	28 (100%)	18.9 ± 6.9	15 (100%)	19.3 ± 7	8 (100%)	17.5 ± 7.1	5 (100%)	20 ± 7.1
	Controls	25 (100%)	9.2 ± 4	8 (100%)	8.7 ± 2.3	10 (100%)	9 ± 4.6	7 (100%)	10 ± 5
	P	-	<0.0001	-	<0.001	-	<0.01	-	0.03
lipase (C14)	RTR	5 (17.9%)	0	0	0	0	0	5 (100%)	5 ± 0
	Controls	0	0	0	0	0	0	0	0
	P	0.05	-	-	-	-	-	0.001	-
leucine arylamidase	RTR	28 (100%)	34.3 ± 10	15 (100%)	32 ± 10.8	8 (100%)	35 ± 10.7	5 (100%)	40 ± 0
	Controls	23 (92%)	28.3 ± 12.8	6 (75%)	19.2 ± 15.9	10 (100%)	32.5 ± 11.8	7 (100%)	30 ± 8.2
	P	0.22	0.01	0.11	<0.01	-	0.52	-	0.02
valine arylamidase	RTR	23 (82.1%)	10.4 ± 9.8	11 (73.3%)	5 ± 0	7 (87.5%)	7.9 ± 5.7	5 (100%)	26 ± 8.9
	Controls	14 (56%)	5 ± 0	2 (25%)	5 ± 0	6 (60%)	5 ± 0	6 (85.7%)	5 ± 0
	P	0.07	<0.01	0.04	-	0.31	0.08	1.0	<0.01
cystine arylamidase	RTR	19 (67.9%)	6.3 ± 3.7	8 (53.3%)	5 ± 0	7 (87.5%)	5 ± 0	4 (80%)	11.2 ± 6.3
	Controls	0	-	0	-	0	-	0	-
	P	<0.0001	-	0.02	-	<0.001	-	0.01	-
a-chymotrypsin	RTR	1 (3.6%)	5	1 (6.7%)	5	0	-	0	-
	Controls	0	-	0	-	0	-	0	-
	P	0.95	-	0.74	-	-	-	-	-
acid phosphatase	RTR	28 (100%)	40 ± 0	15 (100%)	40 ± 0	8 (100%)	40 ± 0	5 (100%)	40 ± 0
	Controls	25 (100%)	35.2 ± 9.2	8 (100%)	40 ± 0	10 (100%)	40 ± 0	7 (100%)	22.9 ± 9.5
	P	-	<0.01	-	1.0	-	1.0	-	<0.01
naphtol-AS-BI-phosphohydrolase	RTR	28 (100%)	30 ± 12.7	15 (100%)	27.7 ± 11.5	8 (100%)	28.1 ± 16.5	5 (100%)	40 ± 0
	Controls	25 (100%)	11.6 ± 8.7	8 (100%)	11.2 ± 9.2	10 (100%)	13 ± 10.1	7 (100%)	10 ± 7.1
	P	-	<0.0001	-	<0.01	-	0.03	-	<0.01
a-galactosidase	RTR	1 (3.6%)	5	1 (6.7%)	5	0	0	0	0
	Controls	0	0	0	0	0	0	0	0
	P	0.95	-	1.0	-	-	-	-	-
b-galactosidase	RTR	3 (10.7%)	5 ± 0	3 (20%)	5 ± 0	0	0	0	0
	Controls	2 (8%)	5 ± 0	0	0	2 (20%)	5 ± 0	0	0
	P	0.89	0.74	0.53	-	0.48	-	-	-
a-glucosidase	RTR	16 (57.1%)	16.6 ± 12.9	15 (100%)	15.7 ± 12.8	1 (12.5%)	30	0	0
	Controls	11 (44%)	6.8 ± 4.6	7 (87.5%)	5.7 ± 1.9	4 (40%)	8.7 ± 7.5	0	0
	P	0.41	0.1	0.35	0.03	0.31	-	-	-
b-glucosidase	RTR	14 (50%)	6.1 ± 2.1	3 (20%)	6.7 ± 2.9	6 (75%)	6.7 ± 2.6	5 (100%)	5 ± 0
	Controls	5 (20%)	6 ± 2.2	0	0	4 (40%)	6.2 ± 2.5	1 (14.3%)	5
	P	0.04	-	0.53	-	0.19	0.81	0.02	<0.01
n-acetyl- b-glucosamidase	RTR	18 (64.3%)	18.1 ± 9.9	15 (100%)	19.7 ± 9.5	3 (37.5%)	10 ± 8.7	0	0
	Controls	13 (52%)	6.9 ± 4.3	8 (100%)	5.6 ± 1.8	4 (40%)	10 ± 7.1	1 (14.3%)	5
	P	0.41	0.03	-	<0.01	1.0	0.88	1.0	0.4
a-mannosidase	RTR	4 (14.3%)	5 ± 0	3 (20%)	5 ± 0	1 (12.5%)	5	0	0
	Controls	0	0	0	0	0	0	0	0
	P	0.11	-	0.53	-	0.44	-	-	-

a Only for active strains, the enzymatic activity is expressed in nmol of hydrolyzed substrates. SD, standard deviation; RTR, renal transplant recipients.

Similar results were achieved when the yeasts species were analyzed solitary. Candida albicans showed higher activity of esterase (C4) (*p*<0.05), esterase lipase (C8) (*p*<0.001), leucine arylamidase (*p*<0.01), naphtol-AS-BI-phosphohydrolase (*p*<0.01) α-glucosidase (*p*=0.03) and n-acetyl-β-glucosamidase (*p*<0.01). Additionally, activity of valine arylamidase and cystine arylamidase was more frequently noted in C. albicans strains from RTR than from controls (Table 1). Candida sp. other than C. albicans isolated from RTR demonstrated higher enzymatic activity of esterase (C4) (*p*=0.02), esterase lipase (C8) (*p*<0.01) and naphtol-AS-BI-phosphohydrolase (*p*=0.03), and were also more frequently positive for cystine arylamidase (*p*<0.001) (Table 1). Strains of Rhodotorula rubra cultured from RTR showed significantly higher activity of esterase (C4) (*p*<0.01), esterase lipase (C8) (*p*=0.03), leucine arylamidase (*p*=0.02), valine arylamidase (*p*<0.01), acid phosphatase (*p*<0.01), naphtol-AS-BI-phosphohydrolase (*p*<0.01) and β-glucosidase (*p*<0.01) compared to strains cultured from immunocompetent subjects. Moreover, following enzymes were also more frequently detected in R. rubra strains achieved from RTR compared to controls: lipase (C14) (*p*=0.001), cystine arylamidase (*p*=0.01) as well as β-glucosidase (*p*=0.02) (Table 1).

## DISCUSSIONS

To aid in the invasion of the host tissues, fungi cells possess constitutive and inducible hydrolytic enzymes that could destroy the host cells (13). Therefore, enzymatic activity of fungi determines their ability to invade different substances and tissues, including keratin, epidermis and dermis. For instance, the aspartic proteases secreted by C. albicans are involved in the adherence process and penetration of tissues, and in interactions with the immune system of the infected host (14). It seems that enzymatic activity of fungi may serve as a pathogenetic marker of these microorganisms. Increased activity of hydrolytic enzymes of Candida strains has been demonstrated in patients with internal neoplasms (15), as well as in subjects with chronic respiratory tract disorders (16). However, to the best of our knowledge, up till now there are no data on enzymatic activity of fungi causing fungal infections in RTR.

Pathogenic fungi, due to long-term immunosuppressive treatment and decreased defense mechanisms in RTR may easily find ideal conditions for growing and invading tissues. The altered function of immune system in RTR may facilitate growth of fungi and could be responsible for the noted higher activity of selected hydrolases. It was observed, that the same strains of fungal pathogenes may demonstrate different enzyme activity depending on the used type of mycotic medium and culture conditions (17-19). The increased enzymatic activity of fungi may probably also determine higher invasiveness of fungal pathogens and higher treatment resistance of onychomycosis in RTR. In conclusion, this study for the first time showed enhanced enzymatic activity of yeast-like fungi isolated from lesional nails in RTR. It is hypothesized that this increased enzymatic activity may be responsible for more aggressive behavior of fungi causing onychomycosis in this group of patients. Further studies are required to clarify completely the observed phenomenon.
